# A New Antiseptic

**Published:** 1870-12

**Authors:** 


### A NEW ANTISEPTIC.

The hydrated chloride of aluminium, to which Mr. John Gambee has recently drawn the attention of medical men and the general public, appears to be a valuable antiseptic. It is quite as potent as chloride of zinc or carbolic acid, and is at the same time non-poisonous, and devoid of unpleasant smell of every kind. These qualities will no doubt ensure its being extensively used, and at no distant date we may expect it to displace the antiseptics which are at present in vogue.

It is somewhat strange that this substance should have been so long overlooked as a possible antiseptic, and Mr. Gamgee certainly deserves credit for suggesting the utilization of it for this purpose. The reason why it has been passed over is probably to be sought in its not being a waste product in any common chemical manufacture. The anhydrous chloride aluminium, which is manufactured in order to serve for the preparation of metallic aluminium, is far too costly on account of the troublesome nature of the process by which it is prepared—to-wit, by passing chlorine at high temperatures over a mixture of aluminium and charcoal. By placing the anhydrous chloride of alumnium in water, it is of course converted into hydrated chloride.

The most economical process for the preparation of the hydrated chloride of aluminium appears to be by double decomposition between sulphate of alumina and chloride of calcium (both of which are cheap commercial products.) When solutions of these two salts are mixed together, sulphate of lime is formed and appears as a precipitate, whilst the hydrated chloride of aluminium remains dissolved.

On allowing the aqueous solution to evaporate at a very gentle heat and afterwards cooling, crystals of hydrated chloride are produced. If an attempt be made to drive off the water from the hydrated chloridé by the application of heat, decomposition will take place. Hydrochloric acid is evolved under these conditions, and oxy-chloride of aluminium is formed, and, by pushing the process, alumina is obtained as the ultimate fixed product.—*Lancet.*

